# Characterization and application of fluorescent hydrogel films with superior mechanical properties in detecting iron(Ⅲ) ions and ferroptosis in oral cancer

**DOI:** 10.3389/fbioe.2024.1526877

**Published:** 2025-01-14

**Authors:** Jinxi Wen, Jian Wang, Siqi Wang, Xingping Zhou, You Fu

**Affiliations:** ^1^ College of Biological Science and Medical Engineering, Donghua University, Shanghai, China; ^2^ Department of General Dentistry, Shanghai Ninth People’s Hospital, Shanghai Jiao Tong University School of Medicine, College of Stomatology, Shanghai Jiao Tong University, National Center for Stomatology, National Clinical Research Center for Oral Diseases, Shanghai Key Laboratory of Stomatology and Shanghai Research Institute of Stomatology, Research Unit of Oral and Maxillofacial Regenerative Medicine, Chinese Academy of Medical Sciences, Shanghai, China; ^3^ Department of Orthodontics, Shanghai Ninth People’s Hospital, Shanghai Jiao Tong University School of Medicine, College of Stomatology, Shanghai Jiao Tong University, National Center for Stomatology, National Clinical Research Center for Oral Diseases, Shanghai Key Laboratory of Stomatology and Shanghai Research Institute of Stomatology, Research Unit of Oral and Maxillofacial Regenerative Medicine, Chinese Academy of Medical Sciences, Shanghai, China

**Keywords:** carbon dots, fluorescent hydrogel film, Fe^3+^, mechanical properties, ferroptosis

## Abstract

A one-step hydrothermal method was applied to prepare carbon dots (CDs) with superior fluorescence properties using chitosan as a carbon source. The as-prepared carbon dots were then grafted onto a sodium alginate-gelatin hydrogel film to form a fluorescent hydrogel film (FHGF), emitting at 450 nm under excitation of 350 nm light. In comparison to the CDs, the fluorescence intensity of this film was maintained over 90.0% and the luminescence position remained basically unchanged, caused by the unchanged surface light-emitting structure of the CDs, due to the existence of electrostatic repulsion between the CDs and the hydrogel. Moreover, the tensile-stress of the fluorescent film with 1.0 wt.% of the CDs was increased by 200% to 10.3 Mpa, and the strain was increased from 117% to 153%. The above experimental results are attributed to the hydrogen bonding between the CDs and the sodium alginate-gelatin hydrogel from analyses of the FT-IR spectra. Interestingly, Fe^3+^ exerted a great quenching effect on this fluorescent film in the concentration range of 0–1.8 μM. The film can be basically used recyclically to detect Fe^3+^ in solution with a detection limit as low as 0.043 μM. In a word, this work demonstrated an enormous potential of carbon dots in fabricating mechanical and fluorescent properties of the hydrogel and proposed a new detection platform for Fe^3+^. In view of the promising Fe^3+^ detection capacity, this hydrogel film can also be applied in oral bacteria surveillance and semi-quantification of ferroptosis in oral cancer.

## Introduction

Hydrogel is a polymer system with a three-dimensional network structure, which has received extensive attention due to its good biocompatibility (Chang et al., 2019) and biodegradability (Hao et al., 2019). Nowadays, hydrogel has been applied in the fields of tissue-engineering (Liu et al., 2020), drug-delivery (Plappert et al., 2019), a portable probe (Li et al., 2024a) and the like. Meanwhile, research on the fluorescent hydrogel is attracting more attention due to its special usages in bioimaging (Mehwish et al., 2019) and environment arraying (Dai and Fidalgo de Cortalezzi, 2019). Scientists have attempted to combine fluorescent materials such as rare-earth compounds (Liu et al., 2019), organic fluorescent dyes (Nishiyabu et al., 2014), and semiconductor quantum dots (Sahiner et al., 2011) with hydrogels to obtain fluorescent hydrogels with some specific properties. However, the above materials usually limit the application range of fluorescent hydrogels due to their toxicity and high cost. Then, with the advent of carbon dots (CDs), scientists have seen ways to solve this problem. They have tried to combine CDs with hydrogels based on the biocompatibility (Ni et al., 2019), low toxicity (Gate et al., 2019) and excellent optical properties (Wang et al., 2010). Besides, the combination of CDs and hydrogels can be widely used in fields such as bioimaging (Zhu et al., 2013) and metal ion probes (Zhang and Chen, 2014). Surprisingly, the addition of CDs can also replace the traditional crosslinkers to enhance the mechanical properties of the hydrogel (Hu et al., 2015). For example, in 2018, Hu et al. (2016) found that adding carbon dots as physical crosslinkers into the polyacrylamide (PAM) hydrogel can greatly improve its mechanical properties. Recently, Wang J. et al. (2018) also found the same phenomenon in synthesizing new p (HEMA-co-AA) fluorescent hydrogels.

Owing to the sensitivity and selectivity of carbon dots to metal ions (Wei et al., 2012), efforts are being made to develop CDs as a completely new metal ion probe. Fortunately, the carbon dots also give the similar performance while being grafted to fluorescent hydrogels (Konwar et al., 2015). Therefore, the use of fluorescent hydrogel as a new ion detection platform has become a new field of exploration (Guo et al., 2017). For instance, in 2018, the PVAm‐g‐N‐CDs/PAM synthesized by Yu et al. (2017) was highly sensitive to Hg^2+^ with a detection limit of 0.089 μM. Moreover, Geng et al. (2015) synthesized fluorescent chitosan hydrogel (3D-FCH), which had a detection function for Hg^2+^ with a very low detection limit. The above all indicated the possibility of fluorescent hydrogels to be a metal ion detection platform.

Generally, iron is an indispensable element for human body and its content in drinking water should be controlled within a certain range. At present, the content of Fe^3+^ in solution is generally determined based on inductively coupled plasma-atomic emission spectroscopy (ICP-AES) and chemical titration. However, the above method is strenuous and cumbersome. Therefore, it becomes very important to explore a new simple method for monitoring iron content. Fortunately, scientists have discovered that Fe^3+^ can cause fluorescence quenching of carbon dots (Qu et al., 2013), implying the possibility of establishing a superior Fe^3+^ detection platform by the use of the CDs-grafted hydrogel. More importantly, from aspect of the promising Fe^3+^ detection capacity, this kind of hydrogel film can also be applied in oral bacteria surveillance and ferroptosis bioimaging (Li X. et al., 2024). Herein, CDs were firstly prepared with excellent fluorescence intensity and then grafted into the hydrogel film. Then, the properties of the composite film were checked including the fluorescence properties and mechanical properties, to investigate effects of the CDs on the film. Importantly, the relationship between the Fe^3+^ concentration and the fluorescence intensity and effects of various metal ions on its fluorescence intensity were also carried out, for exploring the feasibility of fluorescent hydrogels film as a Fe^3+^ detection platform and accelerating the application of CDs, especially in application of oral bacteria surveillance and ferroptosis bioimaging.

## Materials and methods

Chitosan (deacetylation degree ≥95%, viscosity 100 ∼ 200 mpa٠s), gelatin (glue strength ∼240 g Bloom), and sodium alginate (viscosity 200 ± 20 mpa٠s) were purchased from Aladdin. Glycerol (Purity ≥99.0%) was purchased from Greagent and quinine sulfate (purity ≥99%) was purchased from Acros. Salts of FeCl_3_·6H_2_0, Ni (NO_3_)_2_·H_2_O, MgSO_4_·7H_2_0, Ca (NO_3_)_2_·4H_2_O, KCl, Pb (NO_3_)_2_, ZnSO_4_·7H_2_O, AgNO_3_, NH_4_Cl, CuSO_4_·5H_2_0, and EDTA were purchased from Sinopharm Chemical Reagent Co., Ltd., China. None of the above chemicals were further purified. The deionized water used was supplied by the lab.

### Synthesis of carbon dots (CDs)

Chitosan of 1.0 g was dissolved in deionized water of 50 mL, and the resultant was transferred to a high-pressure hydrothermal reaction vessel in an oven, followed by aging at 200°C for 6 h. After the treatments of filtration, centrifugation, dialysis and freeze-drying, brown yellow powder of carbon dots was obtained as received.

### Preparation of fluorescent hydrogel film (FHGF)

Sodium alginate of 2.0 g and gelatin of 0.5 g were dissolved in deionized water of 50 mL and then stirred at 50°C for 4 h. After that, glycerol of 3.0 mL and a certain amount of carbon dots were added to the above solution and then the mixture was agitated at room temperature for 4 h. Hereafter, the resulting suspension was poured into a mold and placed in an oven at 60°C, maintained for 24 h to obtain a dried gel film. Finally, a calcium chloride solution (5.0 wt.%) prepared in advance was sprayed on the surface of the film. After crosslinking for 10 min, the composite film was peeled off to be as a fluorescent hydrogel film (FHGF).

### Cycling experiment of FHGF

The FHGF was soaked in 10–5 M Fe^3+^ solution for 5 min, and then rinsed for 3 times with deionized water to remove the Fe^3+^ remained on the surface. After that, it was further immersed in 10–5 M EDTA solution for 5 min, and then rinsed for 3 times with deionized water. The fluorescence intensity of the FHGF was then measured. The above steps were repeated for 5 times.

### Characterizations

X-ray diffraction (XRD) of CDs was measured using a D/max-2550 PC X-ray powder diffractometer. Optical properties of CDs and FHGF were analyzed using a V-530 UV-visible spectrophotometer and an F-4500 fluorescence spectrophotometer. To obtain material composition information, Fourier transform infrared (FT-IR) spectra of samples were obtained on an Aavatar-380 FT-IR spectrometer. The zeta potential of the samples was analyzed by using the Zestasizer Nano ZS ZEN3600 (Japan). In addition, the surface morphology and structure of the FHGF were analyzed on a Hitachi S-4800 scanning electron microscope (SEM) at an accelerating voltage of 10 kV, and on a JEM-2100 microscope transmission electron microscope (TEM).

### Malondialdehyde (MDA) assay

Ferroptosis was induced by erastin (Absin, China) in the oral squamous cell carcinoma cell line HN4. MDA concentration was measured using MDA Assay Kit (Beyotime, China) following the manufacture’s instruction.

## Results and discussion

### Preparation and characterization of carbon dots (CDs)

**FIGURE 1:**
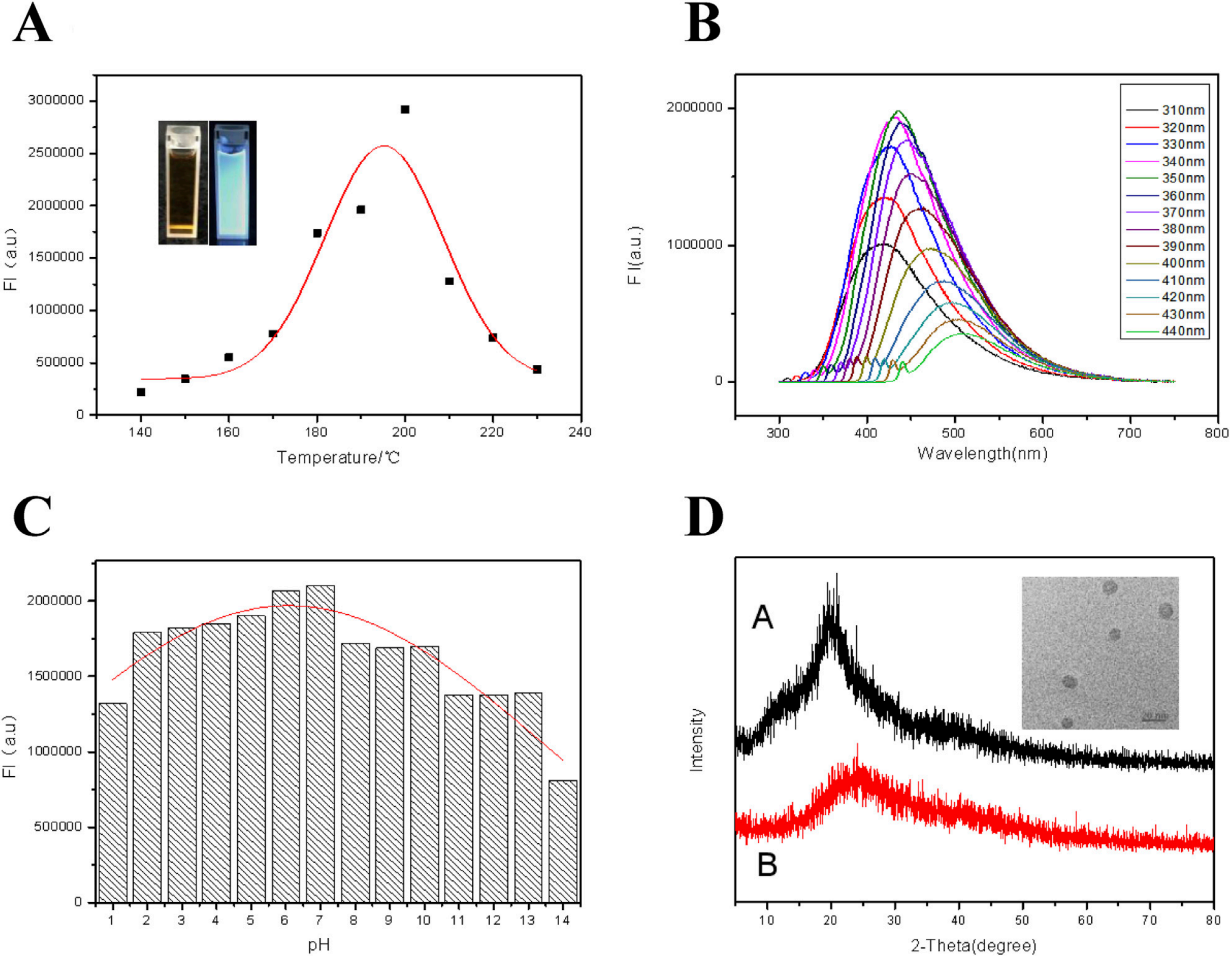
**(A)** Fluorescence intensities of CDs at different reaction temperatures when excited by ultraviolet light of 350 nm. Inset: From left to right are photos of the CDs suspension in natural light and 365 nm UV light. **(B)** Fluorescence emission spectra of the CDs under excitation with different wavelengths. **(C)** Effect of solution pH on fluorescence intensity of the CDs. **(D)** XRD patterns of chitosan (Curve A) and the CDs (Curve B). Inset: TEM image of the CDs.

As shown in Figure 1A, based on the luminescing ability, the optimized reaction temperature for synthesizing carbon dots was 20°C with the chitosan concentration of 2.0 wt.% and the reaction time of 6 h. From the inset of Figure 1A, the CDs suspension appeared dark brown when exposed to sunlight, and appeared bright blue when illuminated with 365 nm UV light. Figure 1B shows the fluorescence spectra of CDs under different excitation wavelengths. It can be easily seen that, when the excitation wavelength moved from 310 nm to 440 nm, a red shift (Qu et al., 2015) occurred in the emission peak. While the ultraviolet light excitation wavelength was 350 nm, the strongest emission peak appeared at 450 nm. The fluorescence of the CDs was also checked in suspension with different pH and the results were shown in Figure 1C. Obviously, in the pH range of 2.0 and 10.0, the fluorescence intensity was basically kept constant. However, the strong acidic or alkaline environment exerted a significant effect on the fluorescence intensity of the CDs, probably resulted from by the broken exiting group on the surface of the CDs under strong acidic and alkaline conditions (Jia et al., 2012). On the other hand, XRD pattern of the CDs was displayed in Figure 1D, together with that of chitosan. The chitosan possessed crystallization characteristics and the CDs were mainly in the form of amorphous carbon, displaying their relatively high purity. Besides, according to the TEM imagine inserted in Figure 1D, the particle with an average diameter of 8.3 nm was spherical with a narrow size-distribution. In a word, the synthesized CDs have good optical properties with good acid and alkali resistances.

### Composition and characterization of FHGF

**FIGURE 2:**
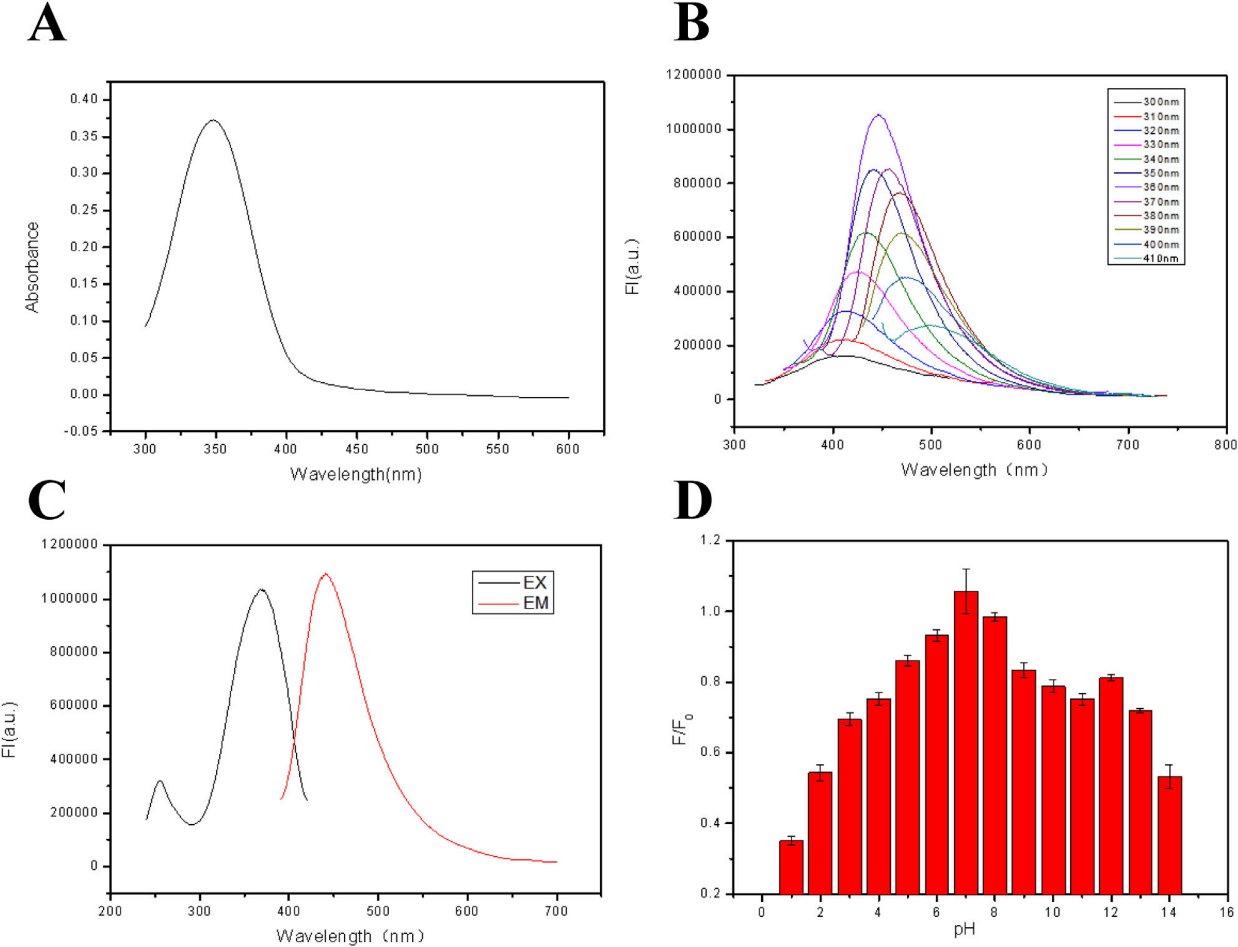
**(A)** UV-vis absorption spectra of the FHGF. **(B)** Excitation and emission spectra of FHF (λ_EX_ = 365 nm, λ_EM_ = 450 nm). **(C)** Fluorescence emission spectra of FHF under excitation with different wavelengths. **(D)** Effect of solution pH on the fluorescence intensity of FHGF. All of the samples were excited at 350 nm.

The optical properties of the fluorescent hydrogel are shown in Figures 2A–C. From Figure 1A, a strong UV absorption peak appeared at 345 nm, probably being attributed to the n–π* transition of a C=O bond (Baker and Baker, 2010). In Figure 2B, when the excitation wavelength was altered from 300 nm to 410 nm, the emission peak position of the film moved from 410 nm to 510 nm. Meanwhile, a red shift phenomenon of the fluorescent hydrogel occurred due to the “Stoke shift,” proving the down-converting luminescence (Ren et al., 2017) property of the film. The dependence of the fluorescent properties on the excitation wavelength is attributed to the naturality of these carbon dots. The fluorescence excitation and emission spectra of the fluorescent film are displayed in Figure 2C. From this figure, when the excitation wavelength is 345 nm, a strong emission peak can be observed at about 450 nm. In addition, the effect of solution pH on the fluorescence intensity of fluorescent films was also explored and the results are revealed in Figure 2D. It is found that when the pH is between 5.0 and 13.0, the fluorescence intensity of the film is relatively strong, but in the strong acid environment, the fluorescence intensity of the film is reduced. This is due to the fact that sodium alginate can react with proton in an acidic environment to form water-insoluble alginic acid, adhering to the surface of the fluorescent film to reduce its fluorescence intensity. The reaction equation is as follows:

NaAlg+HCI→Halg↓+NaCI

**FIGURE 3:**
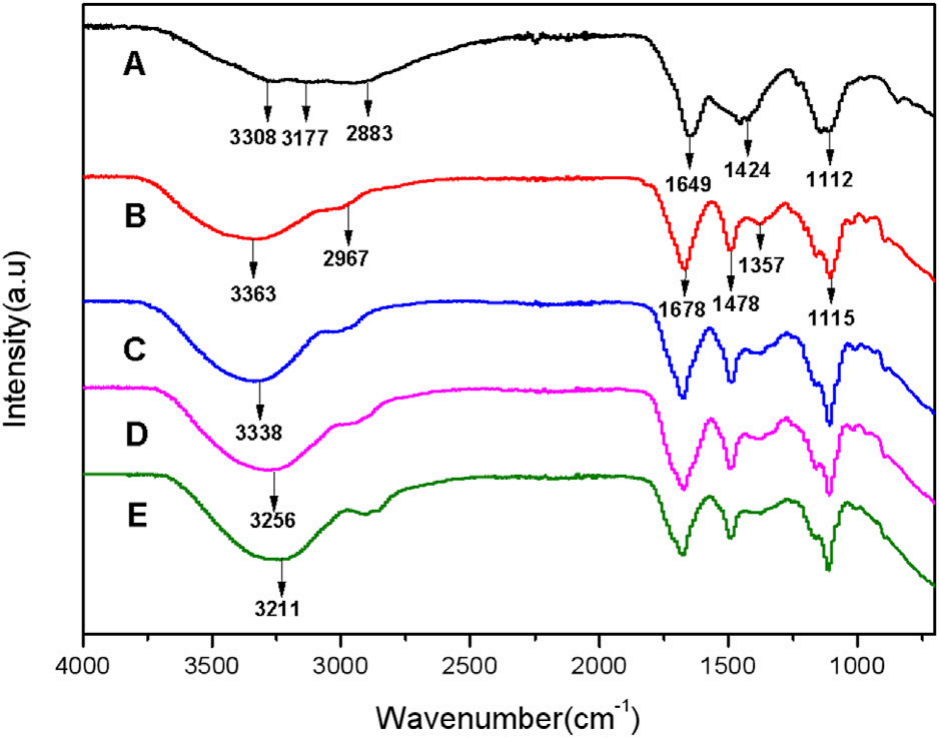
FT-IR spectra of CDs **(A)**, hydrogel films without grafted CDs **(B)**, FHGF with 1.0 wt.% CDs **(C)**, FHGF with 5.0 wt.% CDs **(D)**,and FHGF with 15 wt.% CDs **(E)**.

Figure 3 shows FT-IR spectra of the CDs and the hydrogel films with and without CDs to characterize the structure and composition. The characteristic absorption bands of -OH at 3,308 cm^−1^ and -NH at 3,177 cm^−1^ of the CDs are obtained in Curve A, indicating the presence of an amide group on the surface of the CDs (Fan et al., 2019). The peaks at 2,883 cm^−1^, 1,649 cm^−1^, 1,424 cm^−1^, and 1,112 cm^−1^ are attributed to the stretching vibration of -CH, C=O, -CH_2_^−^, and -C-O-C- (Zhang et al., 2015; Wang et al., 2014), respectively. Besides, Curve B shown in Figure 3 presents FT-IR spectrum of the hydrogel film (HGF) without CDs. HGF has strong and broad peaks at 3,363 cm^−1^, probably caused from the characteristic absorption bands of -OH and -NH-. The peaks at 2,967 cm^−1^, 1,678 cm^−1^, 1,478 cm^−1^, 1,357 cm^−1^ and 1,115 cm^-1^ are attributed to the stretching vibration of -CH-, -C=O, -CH_2_-, -C-(CH_3_)_3_, and -C-O-C- (Sachdev et al., 2016), respectively. Unexpectedly, the peak at 3,363 cm^−1^ in Curve B is significantly red-shifted to the peak at 3,338 cm^−1^ according to Curve C, which shows the FT-IR spectra of FHGF with 1.0 wt.% content of CDs. This indicates the presence of hydrogen bonds between CDs and HGF (Jemmis and Jemmis, 2007). To further confirm the above speculation, the FT-IR spectra of the other two FHGFs containing 5.0 wt.% and 15.0 wt.% CDs were respectively tested and the results were shown on Curves D and E. Obviously, from Curves D and E, as the CDs concentration increased, the red-shifted characteristic absorption band of -NH and -OH became more pronounced, from the peak at 3,256 cm^−1^ to that at 3,211 cm^-1^. In summary, hydrogen bonds were formed between the CDs and the hydrogel film and became stronger along with the increase in CDs content.

**FIGURE 4:**
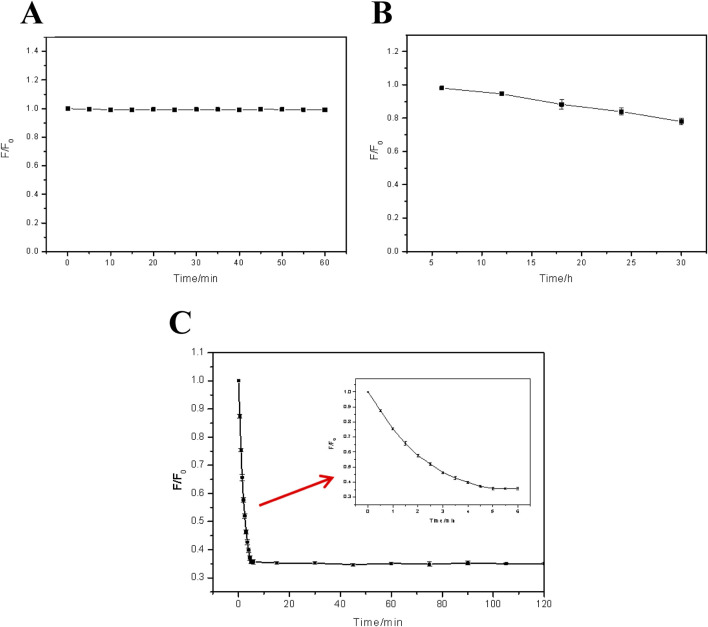
Effect of soaking time on the fluorescence intensity: **(A)** That of FHGF immersed in deionized water for a short time (0min∼60 min); **(B)** That of FHGF immersed in deionized water for a long time (5h∼30 h); **(C)** Relationship between FHF fluorescence and the response time in Fe^3+^ solution with a concentration of 10^–5^ M. All of the samples were excited at 350 nm.

Most working environment of the FHGF is aqueous system, so it is quite important to explore the soaking effect in aqueous environment. Figure 4A indicates the effect of water to the FHGF fluorescence intensity after a short-term immersion. It can be seen from the figure that the aqueous solution does not have a significant effect on the fluorescence intensity of the film in a short time such as less than 1 h, which makes it possible to use the film as a metal ion detection platform. Besides, it can be seen from Figure 4B that when the FHGF is immersed in an aqueous solution for a long time, the fluorescence intensity of the film will be significantly reduced in several 10 h. As the immersion time was 30 h, the fluorescence intensity was decreased by 20%. In a long time, the water molecules can enter the interior of the hydrogel film through the film pores and bind to the CDs in the film by hydrogen bond (Li et al., 2018), and then carry the CDs out of the FHGF, causing the decrease of fluorescence intensity of the film. Figure 4C shows the film soaked in a 10^–5^ M Fe^3+^ solution to investigate the effect of soaking time and determine the optimal reaction time. It can be seen from the figure that the fluorescence intensity of the film markedly decreased in 5 min and remained substantially unchanged while the immersion time exceeded 5 min, indicating that the optimal reaction time was 5 min.

**FIGURE 5:**
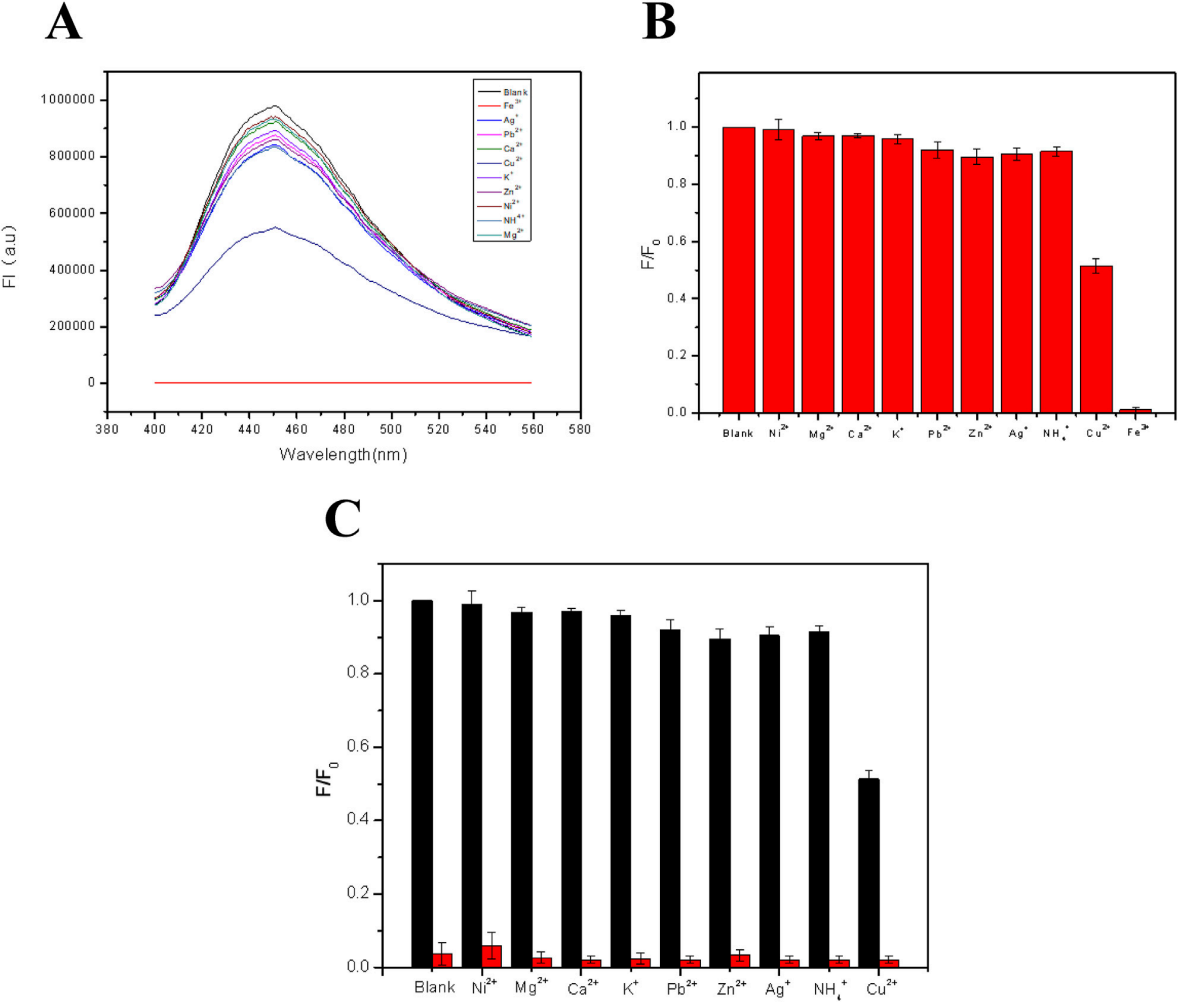
**(A)** Fluorescence patterns of the FHGF soaked in solutions with different metal ions. **(B)** Histogram of the effect of metal ions on the fluorescence intensity of the FHGF. **(C)** Selectivity of the FHGF in the presence of interference cations. All of the samples were excited at 350 nm.

Metal ion selectivity is also a very important factor for detecting metal ions. For checking the selectivity, the fluorescent film was immersed in solutions with different metal ion (Fe^3+^, Ag^+^, Pb^2+^, Ca^2+^, Cu^2+^, K^+^, Zn^2+^, Ni^2+^, NH^4+^, and Mg^2+^) at a fixed concentration of 10^–4^ M. Then, the film was irradiated with a 350 nm UV light for measuring the emission intensities of different samples. It was found from Figures 5A, B that the quenching effect of Fe^3+^ on the fluorescence of the film was mostly obvious, along with a much weaker effect of Cu^2+^, while other metal ions had no obvious effect on the fluorescence. Figure 5C shows the fluorescence intensities of 10^–4^ M Fe^3+^ solution in the presence of different metal ions with the same concentration for checking the anti-interference ability. It has been shown from Figure 5B that the effect of each kind of metal ion on the fluorescence intensity of FHGF before mixing with Fe^3+^ was weak. However, from Figure 5C, with the addition of Fe^3+^, the fluorescence intensity of the film decreased sharply, indicating that the FHGF has unique selectivity to Fe^3+^ and good anti-interference to other metal ions. This is probably due to that Fe^3+^ and CDs in the FHGF can easily form a stable chelate in comparison to the other metal ions with low charges and reduce the fluorescence intensity (Ju and Chen, 2014). In conclusion, the fluorescent film has unique selectivity to Fe^3+^ and good anti-interference to the other metal ions, which is very indispensable for establishing a novel metal ion detection platform.

**FIGURE 6:**
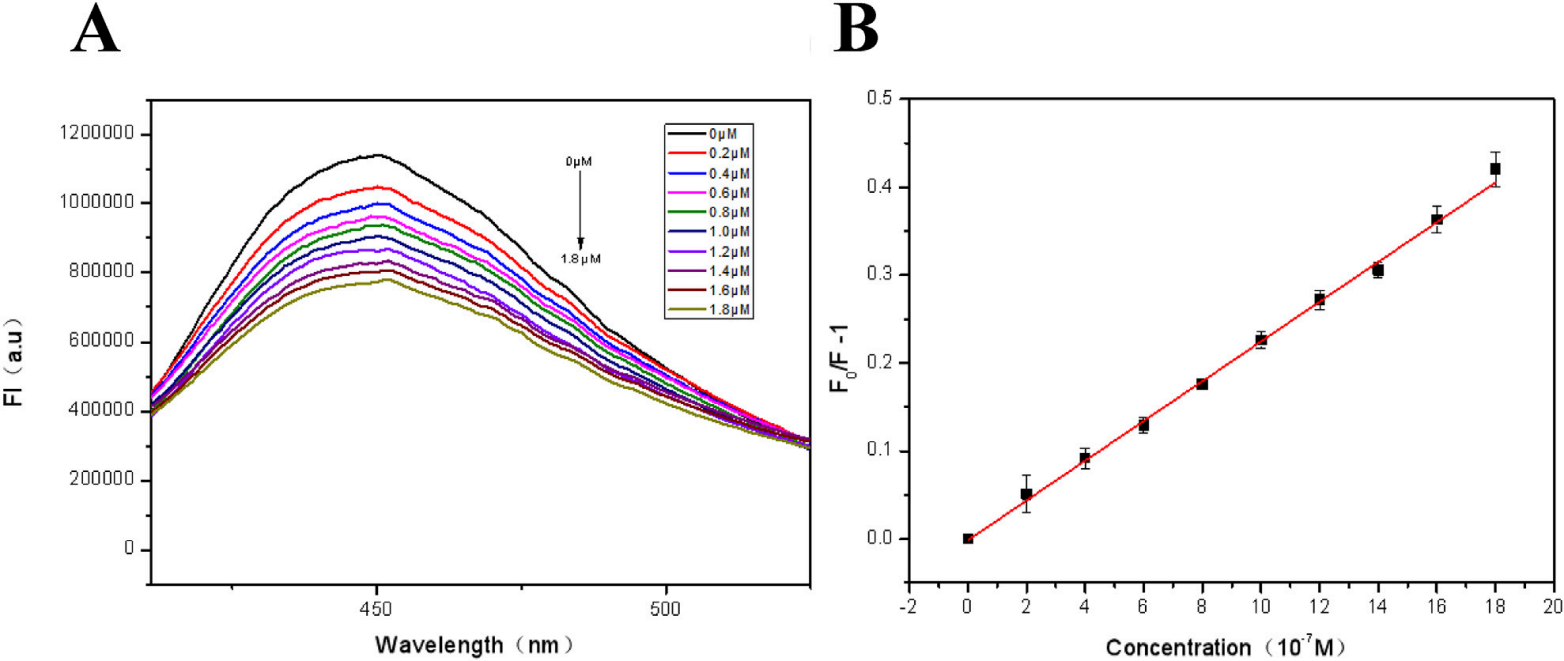
**(A)** Fluorescence spectra of the FHGF at different concentrations of Fe^3+^ (0∼1.8 μM); **(B)** Fluorescence linear equation of FHGF in response to Fe^3+^ concentration. All of the samples were excited at 350 nm.

For the sensitivity study of fluorescent films, Fe^3+^ concentration in the range from 0 to 1.8 μM was selected for investigation. As shown in Figure 6A, the intensity of the FHGF at 450 nm decreased with increasing Fe^3+^ concentration, displaying that there is a negative relativity and this detection system has a good sensitivity to Fe^3+^. Then, the fluorescence quenching were quantifically further analyzed using the Stern-Volmer equation (Friedl et al., 2015).

$$F_{0}/F_{-1}=k_{\text{sv}}C$$

where F0 and F−1 are the fluorescence intensities of the fluorescent film at 450 nm without and with the addition of Fe^3+^ respectively, ksv is the Stern-Volmer quenching coefficient, and C is the concentration of the analyte (Fe^3+^). As shown in Figure 6B, the Stern-Volmer equation exhibited an excellent linear relationship in the concentration range of 0–1.8 μM. The correlation coefficient (R2) = 0.9923, and the detection limit (3σ/s) was 0.043 μM, where σ represents the standard deviation of 10 blank measurements and s is the slope of the calibration curve.

**FIGURE 7:**
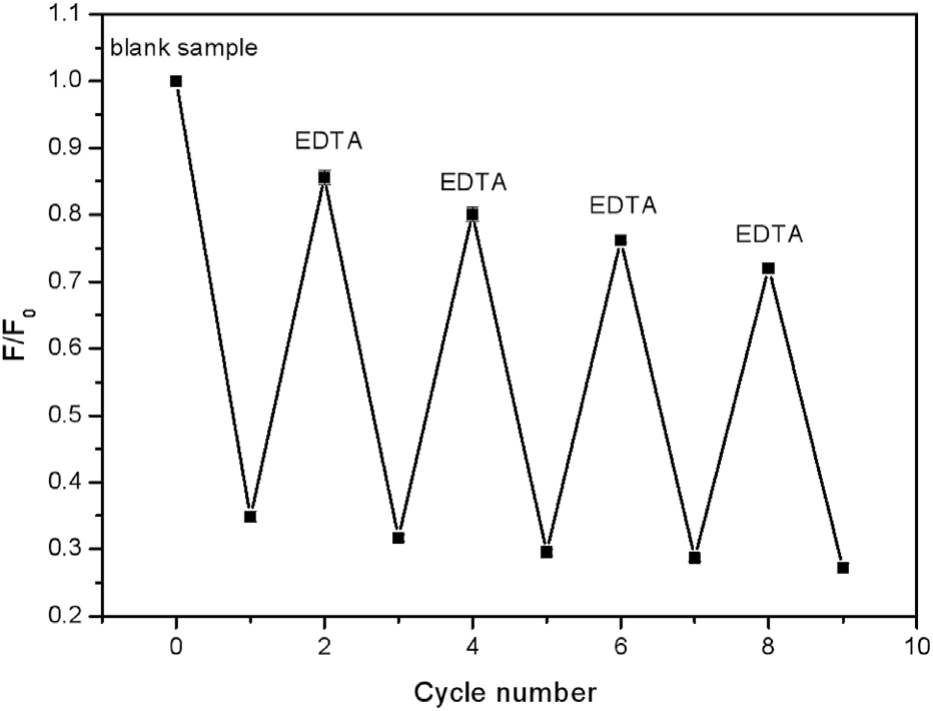
Fluorescence intensity changes of the FHGF after cyclically soaking in Fe^3+^ (10^–5^ M) and EDTA (10^–5^ M) in solution.

The above results have described a potential arraying detection route to Fe^3+^. Then, for the practical usage, repeated application tests are also quite important. To get it, EDTA was used to complex completely with Fe^3+^ ions and then the CDs were released (Wang N. et al., 2018). As shown in Figure 7, when the FHGF was soaked in a 10^–5^ M Fe^3+^ solution, the fluorescence intensity of the FHGF was found to drop sharply. After the addition of EDTA (10^–5^ M), the fluorescence increased significantly, although it could not be restored completely to the original level. Besides, the fluorescence intensity of the FHGF was reduced gently from the previous one after each time EDTA was used. In a word, after four cycles, the FHGF still maintained 76% of the original fluorescence intensity, indicating that this novel platform for detecting Fe^3+^ concentration can be basically used recyclically.

**FIGURE 8:**
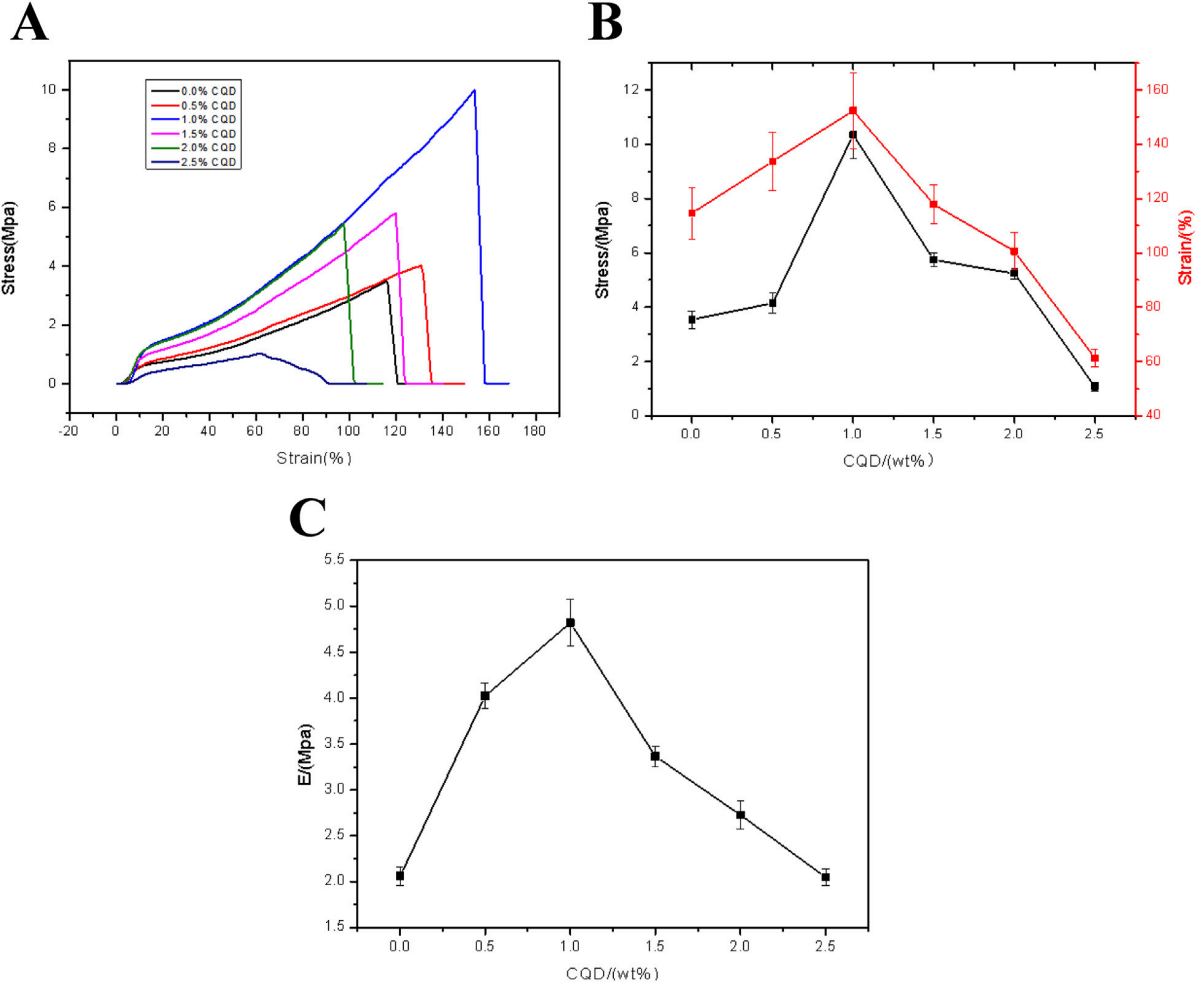
**(A)** Tensile strain-stress curve of the FHGF with different CDs content. **(B)** Stresses and strains of the FHGF as a function of the CDs content. **(C)** Relationship between Young’s Modulus of the FHGF and CDs content.

For the practical application of the FHGF, the mechanical properties are also important. To investigate the effect of CDs on the mechanical properties of the fluorescent films, stress and strain tests at different carbon dots content were conducted. As shown in Figure 8A, the stress and strain of the FHGF increased firstly and then decreased with the addition of CDs, exhibiting an obvious influence from the CDs on the mechanical properties of the FHGF. When the carbon dots content was increased from 0% to 1.0%, the tensile-stress is increased by 200% to 10.3 Mpa and the tensile-strain strength was increased by 36%–152%, as displayed in Figure 8B. However, when the CDs content increased from 1.0 wt.% to 2.5 wt.%, the mechanical properties of the film declined, proving that the mechanical properties of the film were optimal at 1.0 wt.% of the CDs. Additionally, from Figure 8C, when the content of CDs was increased from 0 to 1.0 wt.%, the Young’s modulus of the film was increased by 150% to 4.8 MPa, and then decreased with the increase of CDs content. In order to explain this phenomenon, the Zeta electrical potentials of the CDs and the film in the mixed solution were determined, and the data were listed in Table 1. According to this table, the Zeta potential value of the CDs was −8.18 mV, and that of the film in the mixed solution was −4.57 mV. This indicates that they all have negative charges and they will be repulsive each other. Combined with the previous FT-IR data, it is suggested that due to the hydrogen bond action between the CDs and FHGF, the mechanical properties of the film are enhanced as the content of CDs increases. However, when the content of the CDs is more than 1.0 wt.%, the repulsive effect between them is greater than the connection effect from the hydrogen bond action, resulting in the weakened mechanical properties of the film in this case.

**TABLE 1:** Zeta potentials of CDs and Chitosan- algin.

Sample	Zeta potential value (mV)
CDs	−8.18
Chitosan- algin solution	−4.57

**FIGURE 9:**
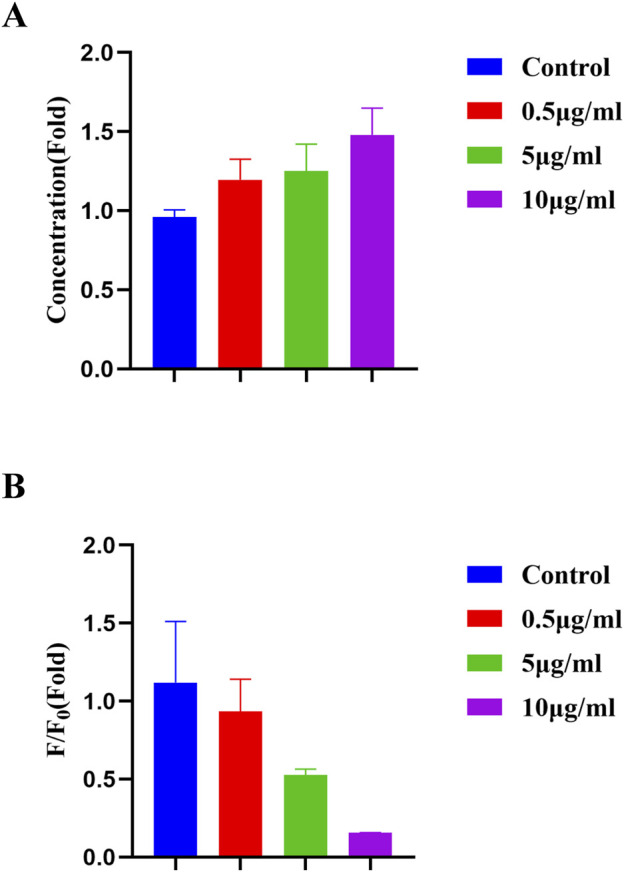
**(A)** MDA concentration of HN4 cells after erastin induction. **(B)** Fluorescence intensity of the FHGF incubated with HN4 cells after erastin induction.

Ferroptosis is a novel form of programmed cell death resulting from iron-dependent accumulation of lipid peroxides. The mechanism of ferroptosis includes disorder of iron metabolism, imbalance of amino acid antioxidant system, and accumulation of lipid peroxides. Ferroptosis was proved to be related to several diseases, such as stroke, tumor, degenerative diseases and cerebral hemorrhage. It was reported that CDs was utilized to induce ferroptosis of tumor cells and enhance antitumor immunity (Li et al., 2024c; Liu et al., 2024). CDs were also applied to observe and inhibit oral bacterial biofilm formation due to its good biocompatibility and bioimaging capacity (Yao et al., 2022). Fe^3+^ usually binds to transferrin and enters into the cells through the transferrin channel, and then participates in a variety of subsequent biochemical processes (Wang et al., 2021). Therefore, Fe^3+^ is an important indicator to determine the occurrence of ferroptosis. Ferroptosis was induced in oral squamous cell carcinoma cell line HN4 by erastin (Guang et al., 2024). Malondialdehyde (MDA) was reported to be a biomarker for ferroptosis (Jiang et al., 2023; Kong et al., 2023). In Figure 9A, MDA concentration exhibited a dose-dependent increase in HN4 cells. Accordingly, because the concentration of Fe^3+^ increased during ferroptosis, the fluorescence intensity of the film decreased also following a dose-dependent way in Figure 9B. In this study, the hydrogel synthesized was of a promising property for Fe^3+^ detection, leading to an ideal tool for ferroptosis tracing and ferroptosis-based therapy.

**FIGURE 10:**
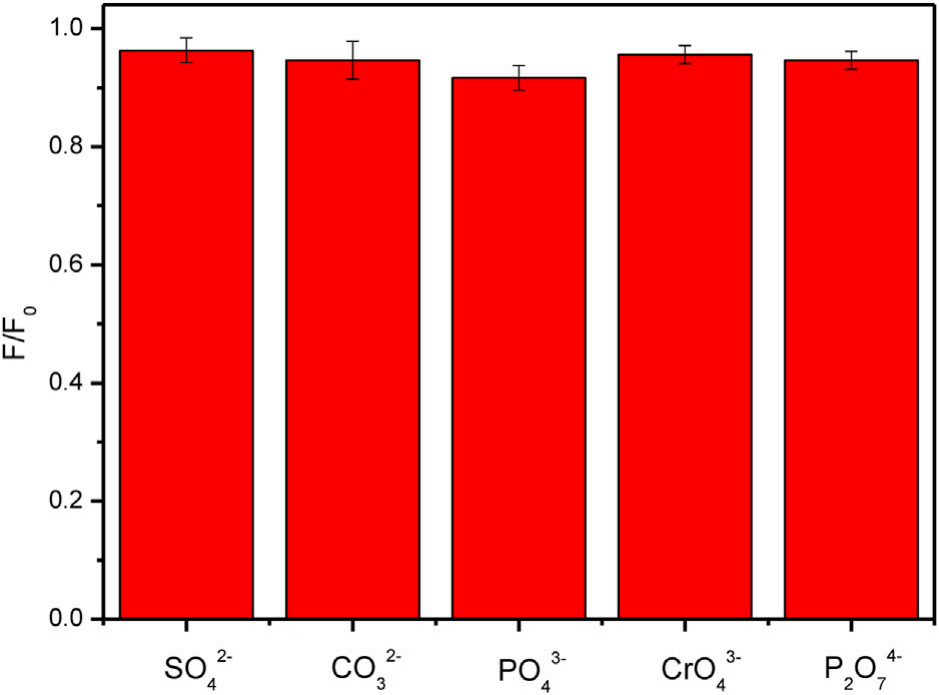
Histogram showing the effect of anions on the fluorescence intensity of the FHGF.

From the above discussion, it is revealed that the surface charges of CDs and the relative materials are a key factor to affect the properties of the composite materials doped with the CDs. Although there have been a lot of reports on the research of CDs composites, the fluorescence or some other properties of the composite materials are not satisfied. In this work, the fluorescent properties of the FHGF were well maintained, which was probably attributed to the negative charge of both the carbon dots and the hydrogel. To further confirm the above idea, the effect of various anions on the fluorescence intensity of the FHGF was tested. As shown in Figure 10, when the FHGF was immersed in an anion solution with the same concentration (10^–4^ M) of SO_4_^2−^, CO_3_^2−^, PO_4_^3−^, CrO_4_^2−^, and P_2_O_7_^4-^, respectively, the fluorescence intensity of the FHGF was substantially unchanged. The above phenomenon indicates that these anions do not have much influence on the fluorescence performance of the film. This further proves the fact that the CDs in the film and ions with the same charge will repel each other, leading to that the light-emitting structure on the surface of the carbon dots is not destroyed and then the fluorescence performance of the CDs is well maintained.

## Conclusion

In this paper, carbon dots (CDs) with good optical properties were synthesized by a one-step hydrothermal method and then grafted into the hydrogel successfully, maintaining their basic optical properties. This is due to the presence of electrostatic repulsion between the CDs and hydrogel, which does not alter the surface morphology of the carbon dots after recombination. Besides, FHGFs obtained by using CDs as crosslinker, not only served as a kind of fluorescent probe for detecting Fe^3+^ content in solution but also had excellent mechanical properties. The synthesized FHGFs are of good sensitivity and selectivity to Fe^3+^ in aqueous solution, achieving that the detection limit of the obtained fluorescent probe is as low as 0.043 μM. The reason for the selectivity is mainly attributed to the positive charge of Fe^3+^ ions, altering the surface charge of negatively charged CDs and thus quenching CDs. Further, the mechanical properties of the film are obviously promoted, due to the comprehensive action of hydrogen bonding and electrostatic interaction between the CDs and hydrogels. The above testing indicates that the addition of CDs greatly enhances the mechanical properties of FHGF and will broaden their application range. Previous studies also demonstrated that CDs could be utilized for oral bacterial biofilm observing and ferroptosis inducing. Most importantly, the results have made it possible to detect Fe^3+^ in solution more conveniently and demonstrate the feasibility of carbon dots as crosslinker. In addition, this hydrogel film could provide a promising strategy for identification of ferroptosis in oral cancer and ferroptosis-based therapy.

## Data Availability

The raw data supporting the conclusions of this article will be made available by the authors, without undue reservation.
